# The relationship among supervisor support, academic passion, academic engagement, and critical thinking in Guangdong tourism management graduate students

**DOI:** 10.3389/fpsyg.2026.1750840

**Published:** 2026-02-03

**Authors:** Xiaomei Cai, Jie He, Xiaodan Zou, Xinni Peng, Tingting Feng

**Affiliations:** 1 School of Economics and Management, South China Normal University, Guangzhou, China; 2 School of Management, Guangzhou University, Guangzhou, China; 3 School of Tourism Management, South China Normal University, Guangzhou, China

**Keywords:** academic engagement, academic passion, critical thinking, graduate students, supervisor support

## Abstract

Critical thinking is essential for individual creativity, professional competence, and the competitive advantage of graduate students. While supervisors are recognized as pivotal in cultivating this skill, the mechanisms underlying their influence remain unclear. Grounded in social information processing theory, this study examines the direct effect of supervisor support on graduate students’ critical thinking, as well as the sequential mediating roles of academic passion and academic engagement. Survey data from 494 graduate students, analyzed using hierarchical regression, reveal that supervisor support positively enhances critical thinking. Moreover, academic passion and engagement sequentially mediate this relationship, whereby supervisor support fosters greater academic passion, which in turn strengthens academic engagement, ultimately improving critical thinking. These findings contribute to theoretical understanding of the pathways linking supervisory support to critical thinking and offer practical implications for enhancing graduate education through targeted supervisory strategies.

## 1 Introduction

Graduate education serves as the primary means of cultivating high-level talent and is a benchmark for the quality of higher education (Aston, 2023; Zhang et al., 2022). Critical thinking is an essential component of graduate education quality (Hart et al., 2021), particularly for management students (Calma and Cotronei-Baird, 2021; Cruz et al., 2021). It refers to rational and reflective thinking skills involved in examining, judging, and adjusting one’s view based on evidence and appropriate standards (Ab Kadir, 2018; Horikami and Takahashi, 2022). In the context of management education, this ability entails analyzing information, drawing reasoned conclusions, and constructing arguments to make informed decisions (Crilly, 2026; Dunn et al., 2014). For graduate students, developing critical thinking skills is crucial for overcoming cognitive biases and thinking constraints (Alvarez-Huerta et al., 2023; Simonovic et al., 2023; Thomas, 2009). As a critical antecedent of creativity, these skills are pivotal to enhancing students’ academic performance (Boryczko, 2022; D’Alessio et al., 2019; Geng et al., 2024) and professional development (Cruz et al., 2021). Consequently, fostering these skills has become a central focus for both educational research and practice.

Research indicates that graduate students’ critical thinking is shaped by both individual characteristics and systemic educational factors (Cruz et al., 2021). Studies of individual characteristics have focused on variables such as age, motivation, self-awareness, and personal belief (Aston, 2023; Lijie et al., 2024). Research on systemic factors has examined elements like the broader education system and curriculum design (Ab Kadir, 2018; Calma and Cotronei-Baird, 2021; Geng et al., 2024). However, supervisor-related factors—particularly the supervisor-student relationship—have been relatively underexplored. As a key factor complementing individual and institutional influences, supervisors play a significant role in the development of graduate students’ competencies (Han et al., 2022; Han et al., 2024).

Recent studies have emphasized the growing importance of mentorship in developing graduates’ research capabilities (Cao et al., 2024; Han et al., 2024; Li et al., 2025). As the cornerstone of graduate education, the supervisor responsibility system places supervisors at the center of graduate students’ academic development. Given that supervisors exert the most direct influence on graduate students, their support constitutes a crucial relational antecedent of student capability development (Anttila et al., 2023; Han and Wang, 2024; Wang et al., 2023). Therefore, a deeper exploration of how supervisor support shapes students’ critical thinking can further clarify and enhance the supervisor’s role in fostering graduate student growth.

Supervisor support refers to the multifaceted assistance and resources that graduate students receive through their interpersonal relationship with their supervisor. This support encompasses academic, personal, and autonomy-related dimensions (Han et al., 2022; Li et al., 2025) and reflects both the tangible resources available to students and the quality of the harmonious interactions within the supervisory dyad (Overall et al., 2011; Vekkaila et al., 2018; Wollast et al., 2023). From a relational perspective, such support is vital for developing of graduate student skills and abilities (Anttila et al., 2023; Nielsen et al., 2017). However, more empirical evidence is required to specifically investigate its impact on the development of graduate students’ critical thinking.

In addition, the mediating processes linking supervisory support to the development of critical thinking in graduate students have yet to be fully elucidated. Critical thinking, as a higher-order cognitive capability, emerges through a constructive process (Boryczko, 2022; Flores et al., 2012; Tuzlukaya et al., 2022). For these students, supervisory support constitutes their most immediate social environment, which shapes their cognitive capabilities through experiential learning and behavioral development (Han and Wang, 2024; Zhang et al., 2022). To understand this linkage, social information processing theory provides a relevant framework. It posits that an individual’s proximal social environment influences their outcomes by shaping their attitudes and behaviors (Kelemen et al., 2025; Zalesny and Ford, 1990). This perspective highlights how individuals adjust their attitudes and behaviors in response to social environments, which in turn shapes their outcomes (Boekhorst, 2015; Kelemen et al., 2025). Therefore, to explain attitudinal and behavioral development, one must account for the individual’s social environment. Applying this logic from social information processing theory, we posit that supervisor support influences graduate students’ critical thinking by shaping their learning-related attitudes and behaviors. More specifically, we propose that academic passion and engagement serve as key mediators in the relationship between supervisor support and the development of critical thinking in graduate students.

**Figure 1:**
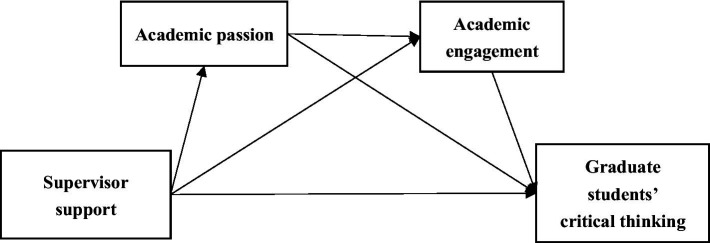
The conceptual model.

Academic passion refers to the attitudes of graduate students toward their academic pursuits, including affective, cognitive, and volitional dimensions. It serves as an intrinsic motivator for the development of thinking skills (Izadpanah, 2023). Academic engagement refers to students’ behavioral participation in academic activities, characterized by sustained energy, mental vigor, and cognitive absorption in learning (Alemayehu and Chen, 2023; Robayo-Tamayo et al., 2020). As a proximal determinant of critical thinking skills, academic engagement thus functions as a key mediating mechanism through which supervisor support enhances graduate students’ critical thinking (Crilly, 2026; Tseng et al., 2020). Empirical evidence further indicates that academic passion positively influences academic engagement, thereby fostering deeper and more sustained academic involvement (Stoeber et al., 2011; Zhao et al., 2021). Building on this relationship, we propose a sequential mediation model in which academic passion and engagement serially transmit the influence of supervisor support to critical thinking. In such a model, supervisor support affects critical thinking through academic passion and engagement (Zugna et al., 2022). The conceptual model is shown in Figure 1.

This study attempts to make three contributions to the literature. First, it identifies supervisor support as a predictor of graduate students’ critical thinking, thereby extending our understanding of thinking capability development within the supervisory relationship. Second, drawing on social information processing theory, it elucidates the psychological mechanism of this relationship by testing the sequential mediation of academic passion and engagement. Third, it provides empirical evidence for the application of this theory in graduate education contexts.

### 1.1 Supervisor support and graduate students’ critical thinking

Critical thinking is conceptualized as an individual’s capacity for reasoned judgment and rational decision-making (Crilly, 2026; Hammer and Green, 2011). This capacity involves the skills of understanding, analyzing, reasoning, and evaluating, as well as dispositions such as open-mindedness that facilitate effective problem-solving (Lee et al., 2026; Miri et al., 2007). Supervisor support, a key relational factor in graduate education, encompasses both resources and supportive behaviors provided to the student (Cohen et al., 2000; Li et al., 2025). These resources include constructive feedback, opportunities for research engagement, and emotional support (Han et al., 2022; Vekkaila et al., 2018), while supportive behaviors involve encouraging students to voice their perspectives and motivating them to complete tasks (Govaerts and Dochy, 2014; Han and Wang, 2024). Commonly, supervisor support is categorized into four types: instrumental, emotional, informational, and co-constructional support (Anttila et al., 2023; Sagasser et al., 2015).

In graduate education, the supervisor-student relationship plays a pivotal role in fostering critical thinking. It facilitates a socialization process through which students dynamically develop their abilities in comprehension, analysis, inference, and evaluation. As the most direct source of social support for graduate students, supervisor support enhances critical thinking by providing both motivational and developmental opportunities (González-Ocampo and Castelló, 2019; Zhang et al., 2022). This occurs in two primary ways. First, through academic guidance and informational support, supervisors enhance students’ domain knowledge, self-efficacy, and overall academic experience (Robayo-Tamayo et al., 2020; Zhang et al., 2022). Second, through supportive interactions, supervisors stimulate students’ academic interests and strengthen their decision-making capacity when solving problems (Anttila et al., 2023). Empirical evidence indicates that effective supervisors foster critical thinking by motivating students and creating opportunities for its development (Lee, 2008; Shahab and Barak, 2026). This cognitive skill is cultivated through systematic engagement in exploration, analysis, and problem-solving (Crilly, 2026; Şendağ and Ferhan Odabaşı, 2009). Specifically, supervisors guide students to develop rational thinking patterns, make reflective adjustments, and sustain this process through academic challenges. Based on this reasoning, we propose the following:

*H1*: Supervisor support has a positive impact on graduate students’ critical thinking.

### 1.2 The mediating effect of academic passion

Passion involves a persistent emotional orientation toward activities that individual’s value and consistently pursue (Bélanger and Ratelle, 2021). This psychological state manifests as sustained engagement and goal-directed behavior. Contemporary research distinguishes two dimensions of passion: obsessive passion, which is driven by external contingencies, and harmonious passion, which arises from intrinsic motivation. Within academic contexts, passion represents an integrated psychological orientation that combines cognitive, affective, and volitional components toward academic pursuits (Izadpanah, 2023). Harmonious academic passion, in particular, is characterized by a positive and engaged orientation toward intellectual activities such as inquiry and problem-solving. This orientation fosters the development of students’ critical thinking skills (Schellenberg and Bailis, 2017; Sun et al., 2023; Wang et al., 2025).

Academic passion is significantly influenced by supervisor support. Indeed, the formation of positive academic attitudes and experiences fundamentally depends on such support (Han and Wang, 2024). Empirical evidence further indicates that supervisors’ provision of academic guidance and research resources directly fosters the development and maintenance of graduate students’ academic passion (Li et al., 2025). This relationship aligns with self-determination theory, which posits relatedness as one of three fundamental psychological needs essential for human motivation and development (Deci and Ryan, 2008; Manninen et al., 2022).

The supervisory relationship constitutes the most immediate and influential source of social support for graduate students. High-quality mentorship fulfills their fundamental need for relatedness (Deci and Ryan, 2008; Wang et al., 2024), thereby enhancing their academic passion. This passionate engagement, characterized by strong identification with and enthusiasm for research work, in turn cultivates critical thinking skills (Alvarez-Huerta et al., 2023; Ruiz-Alfonso and León, 2016). The research process inherently cultivates these abilities through sustained exploration, analysis, synthesis, and problem-solving. Consequently, as students invest greater cognitive and temporal resources into their work, their higher-order critical thinking capacity improves significantly (Zhou, 2021).

*H2*: Academic passion mediates the relationship between supervisor support and graduate critical thinking.

### 1.3 The mediating effect of academic engagement

Academic engagement refers to students’ active, effortful involvement in learning, characterized by deliberate attempts to comprehend, analyze, and apply knowledge (Alvarez-Huerta et al., 2023; Hospel et al., 2016; Perkmann et al., 2021; Yun and Park, 2020). Within graduate education, supervisor support has a positive impact on students’ academic engagement. Specifically, the provision of academic resources and psychosocial support enhances students’ self-efficacy and fulfills their basic psychological needs, thereby promoting deeper and more sustained learning involvement (Jiang and Tanaka, 2022; Zhang and Kim, 2025). Furthermore, positive supervisory interactions significantly enhance graduate students’ learning motivation.

Supervisor support fosters a constructive learning environment, one that reduces psychological barriers, stimulates intellectual curiosity, and cultivates sustained academic interest. This environment, in turn, promotes deeper cognitive engagement and greater dedication of time to academic pursuits (Fredricks et al., 2016; Wang and Wang, 2024).

Supervisor support enhances graduate students’ critical thinking by fostering their academic engagement. Critical thinking develops as students actively internalize supervisory support into their cognitive competencies through deliberate practice and reflection (Bandura, 1997; Wang and Wang, 2024). This internalization is operationalized through engagement in academic activities, where students encounter diverse perspectives, build domain knowledge, and rigorously exercise higher-order cognitive skills (Loyola-Carrillo et al., 2025; Pekrun et al., 2011). Empirical evidence confirms that engaged learners show significantly greater improvement in analytical and evaluative thinking (Alvarez-Huerta et al., 2023; Fredricks et al., 2016; Miao et al., 2025). Within this context, supervisor support emerges as a pivotal factor in facilitating academic engagement (Cao et al., 2024; Vekkaila et al., 2018), thereby indirectly enhancing both knowledge integration and critical thinking capacities (Santos et al., 2023; Vincent-Lancrin, 2023). Based on this theoretical and empirical foundation, we propose the following hypothesis:

*H3*: Academic engagement mediates the relationship between supervisor support and graduate students’ critical thinking.

### 1.4 The sequential mediating effects of academic passion and engagement

Social information processing theory elucidates how individuals cognitively transform external social cues into attitudinal and behavioral responses that shape their outcomes. According to the theory, this occurs through a sequential three-stage process: attending to and perceiving social cues, forming an evaluative attitude in response, and formulating a corresponding behavior (Kelemen et al., 2025; Zalesny and Ford, 1990). Social information serves as the primary input for this process, driving individuals’ sense making of their environment and guiding their subsequent adaptations. Thus, outcomes are ultimately determined by this mediated chain of perception, attitude formation, and behavior.

Within academic settings, supervisor support is the primary conduit of social information about research norms and expectations (Cao et al., 2024). According to social information processing theory, this support shapes critical thinking development through a sequential mediation process involving academic passion and academic engagement. Specifically, supervisor support fosters academic passion as a proximal outcome (Bonneville-Roussy et al., 2013; Li et al., 2025; Ruiz-Alfonso and León, 2016). This passion, in turn, positively predicts and enhances academic engagement (Stoeber et al., 2011; Zhou, 2021). It is through this heightened engagement that students’ critical thinking skills are ultimately developed and refined (Santos et al., 2023).

Informed by the established link between academic passion, deep engagement, and improved analytical skills, this study proposes a sequential mediation model (Curran et al., 2015; Shahab and Barak, 2026). Specifically, we hypothesize that supervisor support indirectly enhances graduate students’ critical thinking by fostering their academic passion, which in turn increases their academic engagement, thereby developing their critical thinking competencies.

*H4*: Academic passion and engagement play sequential mediating roles between supervisor support and graduate students’ critical thinking.

## 2 Materials and methods

### 2.1 Participants

This study employed a quantitative research method. Between August 8 and 28, 2022, online questionnaires were distributed to graduate students in tourism management using a convenience sampling approach. The respondents were selected for the following reasons: First, the tourism industry is highly dynamic and faces complex challenges such as cross-cultural management, sustainability issues, and unpredictable disruptions. Consequently, tourism management graduate students require well-developed critical thinking skills to systematically evaluate information, assess risks, and generate innovative solutions (Tang et al., 2024; Tzanelli and Korstanje, 2020). Second, the field of tourism management frequently grapples with ethical dilemmas, from over-commercialization to community conflicts. Furthermore, conducting tourism research necessitates integrating diverse disciplinary perspectives, including economics, sociology, and environmental science. Within this complex landscape, critical thinking is an indispensable competency. It allows graduate students to synthesize distinct theoretical frameworks, balance competing stakeholder interests, and ultimately formulate strategies that advance long-term sustainability (Bramwell and Lane, 2014; Tang et al., 2024).

Questionnaires were distributed to graduate students through online WeChat groups. WeChat is a widely used multi-platform instant messaging application in China, which facilitates the creation of groups for administrative purposes such as disseminating notifications, sharing information, and fostering communication among members.

The anonymous questionnaires were distributed to graduate students via WeChat groups, leveraging academic networks and personal connections to reach students at universities across Guangdong Province. A total of 521 questionnaires were returned, yielding 494 valid responses (a 94.8% response rate). Respondent characteristics are summarized in Table 1. To contextualize the sample, universities in China are often categorized by national initiatives. “Project 211,” launched in 1995, aimed to enhance research quality at approximately 100 key universities. Its subset, “Project 985” (1998), focused on developing about 39 of these into world-class institutions (Lin and Wang, 2022).

**Table 1:** The characteristics of samples.

Variables	Items	Frequency	Percentage
Gender	Male	129	26.11%
	Female	365	73.89%
Grade	First year	172	34.82%
	Second year	198	40.08%
	Third year	124	25.10%
Title of supervisor	Professor	265	53.64%
	Associate professor	209	42.31%
	Lecturer	20	4.05%
Level of university	Project 985	45	9.11%
	Project 211	184	37.25%
	Others	265	53.64%

### 2.2 Measures

The questionnaire measured five variables. All items utilized a 5-point Likert scale ranging from 1 (strongly disagree) to 5 (strongly agree).

Perceived supervisor support was assessed using a 12-item scale based on Overall et al. (2011). The use of this scale in a similar context is supported by recent work (Han et al., 2022). This scale included four items pertaining specifically to academic support (e.g., “My supervisor provides practical advice on planning and implementing academic research”). It also measured emotional support (e.g., “My supervisor expresses understanding and empathy when I encounter difficulties”) and autonomy support (e.g., “My supervisor offers opportunities for me to independently choose my academic research direction”). Cronbach’s Alpha was 0.957.

Academic passion was measured using an eight-item scale developed by Vallerand et al. (2007). The Chinese application of this scale follow the work of Zhou et al., (2025). Sample items included “*Engaging in academic research gives me a sense of fulfillment” and* “*If possible, I would devote all my time to academic research.*” Cronbach’s Alpha was 0.912.

The measurement of academic engagement was from Schaufeli et al. (2002). It was measured using the scale developed by Wickramasinghe et al. (2023). There were three items, for example, “*I actively participate in group meetings and discussions within my research team” and “I often discuss academic questions with students from other discipline*.” Cronbach’s Alpha was 0.796.

Critical thinking was assessed using a nine-item scale developed by Facione et al. (1995) and shortened by Bell and Loon (2015), a version that has been applied in relevant educational context (Wickramasinghe et al., 2023). Representative items include: *“I am skilled at solving problems” and “I effectively apply the knowledge I have learned.”* Cronbach’s Alpha was 0.915.

### 2.3 Method

This study employed structural equation modeling (SEM) to investigate the relationships among supervisor support, academic passion, academic engagement, and graduate students’ critical thinking. SEM is a powerful multivariate technique that enables the simultaneous testing of relationships between latent constructs and their observed indicators (Bollen, 1989b; Han et al., 2022).

In this study, confirmatory factor analysis (CFA) was performed using structural equation modeling (SEM) to assess the construct validity of the measurement model, testing how well the observed variables represented their respective latent constructs (Bollen, 1989a; Yang et al., 2025). The application of SEM aligns with its established use in social science research, particularly within education (Tan and Koh, 2023). For instance, SEM is widely adopted in studies that examine complex relationships among factors influencing student academic performance and development, as it provides in-depth insights into such models (Yin and Huang, 2021).

### 2.4 Confirmatory factor analyses

To validate the measurement model, LISREL 8.80 was applied to test the validity of the variables. The CFA results showed that the six-factor model (academic support, emotional support, autonomous support, critical thinking, academic passion, and academic engagement) fit the data well (Table 2). The six-factor model with the ratio of chi square to degree of freedom = 3.32 < 5; Root mean square error of approximation (RMSEA) = 0.097 < 0.1; Normed fit index (NFI) = 0.93; Non-normed fit index (NNFI) = 0.94; Comparative fit index (CFI) = 0.95; Incremental fit index (IFI) = 0.95 showed better than other models. The CFA results also indicated that the common method bias was acceptable in this study (Hu and Bentler, 1999).

**Table 2:** Results of CFA.

Models	*χ*^2^/*df*	RMSEA	NFI	NNFI	CFI	IFI
Single-factor	6408.72/464 = 13.81	0.228	0.70	0.69	0.71	0.71
Two-factors	3524.85/463 = 7.61	0.164	0.83	0.84	0.85	0.85
Three-factors	1943.27/461 = 4.22	0.114	0.91	0.92	0.93	0.93
Four-factors	1860.50/458 = 4.06	0.112	0.91	0.93	0.93	0.93
Six-factors	1491.88/449 = 3.32	0.097	0.93	0.94	0.95	0.95

*N* = 494; Single-factor: supervisor support + critical thinking + academic passion + academic engagement; Two-factors: supervisor support + critical thinking, academic passion + academic engagement; Three-factors: supervisor support, academic passion + academic engagement, critical thinking. Four-factors: supervisor support, academic passion, academic engagement, critical thinking; Six-factors: academic support, emotional support, autonomous support, critical thinking, academic passion, and academic engagement.

### 2.5 Descriptive statistics

Table 3 presents the means, standard deviations, and correlations among the variables. The results reveals that the key variables, including supervisor support, critical thinking, academic passion, and academic engagement are positively correlated with each other.

**Table 3:** Mean, standard deviations and correlations of key variables.

Variables	Mean	SD	1	2	3	4
1. Supervisor support	4.11	0.80	1			
2. Academic passion	3.28	0.78	0.48**	1		
3. Academic engagement	3.51	0.73	0.47**	0.59**	1	
4. Critical thinking	3.90	0.56	0.44**	0.57**	0.59**	1

*p < 0.05, **p < 0.01, ***p < 0.001.

## 3 Results

SPSS 22.0 with Process 3.0 was used to test the hypothesized sequential model (Igartua and Hayes, 2021). The regression results (Table 4) supported the proposed relationships. Specifically, supervisor support had a positive effect on graduate students’ critical thinking (M3: *b* = 0.37, *p* < 0.001), supporting H1. It also positively predicted academic passion (M1: *b* = 0.51, *p* < 0.001) and academic engagement (M2: *b* = 0.46, *p* < 0.001). After controlling for supervisor support, academic passion was positively associated with critical thinking (M4: *b* = 0.36, *p* < 0.001). However, the direct effect of supervisor support on critical thinking was attenuated (M2: *b* = 0.19, *p* < 0.001). This result indicates that academic passion partially mediates the relationship between supervisor support and critical thinking, thereby supporting H2.

**Table 4:** Regression results.

Variables	Academic passion	Academic engagement	Critical thinking	Critical thinking	Critical thinking	Critical thinking
	M1	M2	M3	M4	M5	M6
Constant variable	1.22	2.06	0.78	2.10	1.69	1.65
Control variable						
Gender	−0.08	−0.14	−0.15	−0.12	−0.09	−0.09
Grade	−0.01	0.02	0.01	0.02	0.01	0.01
Title of supervisor	−0.05	−0.03	−0.01	0.03	0.02	0.03
Level of university	0.05	0.00	−0.06	−0.08	−0.06	−0.07
Independent variables						
Supervisor support	0.51***	0.46***	0.37***	0.19***	0.18***	0.12***
Mediator						
Academic passion				0.36***		0.23***
Academic engagement					0.41***	0.30***
*R*	0.49	0.48	0.46	0.61	0.63	0.67
*R* ^2^	0.24	0.23	0.21	0.37	0.39	0.45
*F*	30.67***	28.72***	21.25***	47.79***	52.72***	55.97***

*p < 0.05, **p < 0.01, ***p < 0.001. Regression coefficients between key variables are shown in bold.

Similarly, the results confirmed that academic engagement was positively related to critical thinking (M5: *b* = 0.41, *p* < 0.001) after controlling for supervisor support. H3 was supported.

Furthermore, when academic passion and academic engagement were included as mediators (M6), the coefficient for the direct effect of supervisor support on critical thinking decreased from 0.37 (*p* < 0.001) to 0.12 (*p* < 0.001), and that for academic passion decreased from 0.36 (*p* < 0.001) to 0.23 (*p* < 0.001). In sum, academic engagement mediated the influence of both supervisor support and academic passion on critical thinking.

The sequential mediating effects were examined using nonparametric bootstrapping procedures (Table 5). The analysis revealed a positive total effect of supervisor support on critical thinking (*b* = 0.37, 95% CI [0.30, 0.43]). The direct effect of supervisor support on critical thinking was positive (*b* = 0.12, 95% CI [0.05, 0.18]). The bootstrap test indicated indirect effects for the path via academic passion (*b* = 0.12, 95% CI [0.08, 0.16]), the path via academic engagement (*b* = 0.07, 95% CI [0.04, 0.11]), and the sequential path via academic passion and academic engagement (*b* = 0.06, 95% CI [0.05, 0.09]). Since none of the confidence intervals included zero, supporting H4.

**Table 5:** Results of the mediating effects.

Effects	Path	Coefficient	SE	*t*	*p*	LLCI	ULCI
Direct effect	Supervisor support→critical thinking	0.12	0.03	3.55	0.00	0.05	0.18
	Supervisor support→academic passion→critical thinking	0.12	0.02	—	—	0.08	0.16
Indirect effect	Supervisor support→academic engagement→critical thinking	0.07	0.02	—	—	0.04	0.11
	Supervisor support→academic passion→academic engagement→critical thinking	0.06	0.01	—	—	0.05	0.09
Total effect	Supervisor support→critical thinking	0.37	0.03	11.09	0.00	0.30	0.43

## 4 Discussion

### 4.1 Theoretical implications

Drawing on social information processing theory, this study investigates how supervisor support influences graduate students’ critical thinking by elucidating the underlying mechanisms. This study makes three key contributions to the graduate education literature.

First, it extends critical thinking research by specifically examining its predictor among graduate students, a population with an explicit emphasis on critical thinking skills development (Calma and Cotronei-Baird, 2021; Crilly, 2026; Halpern, 2014; Shahab and Barak, 2026). It builds on mentorship research by demonstrating how the supervisor responsibility system in graduate education acts as a crucial mechanism for competency development. This extends prior theory and provides empirical validation for the pivotal role of supervisory support in fostering critical thinking (Nielsen et al., 2017; Zhang et al., 2022). Furthermore, the results support conceptualizing graduate students’ critical thinking as an advanced, socially-constructed capability that emerges through interactions between supervisor and graduate students (Aston, 2023).

Second, this study advances the literature by examining the impact of supervisor support on graduate students’ critical thinking through a relational lens, a perspective underexplored in existing research. While prior studies have predominantly focused on individual factors (e.g., prior knowledge, motivation) or systemic factors (e.g., curriculum design, institutional support) (Ab Kadir, 2018; Aston, 2023; Boryczko, 2022; Lijie et al., 2024), this investigation shifts attention to mentorship. It specifically elucidates how supervisor support facilitates critical thinking through social-cognitive mechanisms. This finding aligns with established theoretical perspectives that position supervisor support as a critical form of social support, demonstrating significant positive effects on graduate students’ competency development (Cao et al., 2024; Han and Wang, 2024; Li et al., 2025). The impact of supervisory relationships on the development of graduate students’ critical thinking has been historically underemphasized in the literature (Li et al., 2025; Miao et al., 2025). As the primary source in academic settings, supervisor support represents a fundamental yet understudied mechanism for cultivating higher-order thinking skills. The findings substantiate that supervisor support has a positive impact on critical thinking competency, thereby expanding theoretical understanding of cognitive development through a relational lens.

Third, this study contributes to the literature on academic motivation and cognition by elucidating the mechanism linking supervisor support to graduate students’ critical thinking. The findings reveal that academic passion and engagement serve as crucial mediating pathways, thereby explaining the psychological mechanism in the relationship between supervisor support and graduate students’ critical thinking. Grounded in social information processing theory, we provide a novel framework for understanding how supervisor support translates into graduate students’ critical thinking. This extends prior work demonstrating motivational influences on critical thinking (Han and Wang, 2024; Lijie et al., 2024). It highlights that motivation is not given, but arises in a specific social environment (Kelemen et al., 2025; Zalesny and Ford, 1990).

This study conceptualizes critical thinking as the product of a social construction process (Geng et al., 2024). Consistent with this view, graduate students’ critical thinking is motivated by social relationships. As prior research notes, the perception of the social environment serves as an antecedent to individual attitudes and behaviors (Boekhorst, 2015; Miao et al., 2025; Zhang et al., 2022). The findings specify this mechanism by showing how supervisor-transmitted social information is internalized through passionate engagement, thereby facilitating critical thinking. The results confirm a sequential mediation path (supervisor support → academic passion → academic engagement → critical thinking), empirically validating social information processing models within graduate education.

### 4.2 Practical implications

First, within the framework of China’s supervisor responsibility system, supervisors must proactively fulfill their pivotal role by providing comprehensive support. This entails addressing students’ needs in a timely manner, supplying essential resources (e.g., social capital, academic networking opportunities, access to advanced research), and maintaining open lines of communication. Beyond instrumental support, supervisors should offer emotional encouragement and constructive guidance to bolster students’ confidence and intrinsic motivation. Practical measures, such as assisting with research planning and facilitating regular presentations, are effective means to ensure continuous development.

Second, supervisors should actively cultivate graduate students’ academic passion, as it is the intrinsic driver of critical thinking and sustained research engagement. When students encounter difficulties in executing research plans independently, supervisors can provide targeted academic guidance coupled with consistent emotional support to bolster confidence. Furthermore, supervisors should broaden students’ intellectual perspectives through knowledge-sharing, foster genuine curiosity in their research topics, and enhance academic self-efficacy by entrusting them with autonomy while providing supportive assistance.

Third, a key supervisory priority should be to foster graduate students’ academic engagement, given its fundamental role in cultivating critical thinking. Since this higher-order skill is refined through practice, active engagement systematically strengthens students’ ability to analyze and solve problems. This requires students to adopt proactive academic attitudes and behaviors. In essence, academic engagement serves as the crucial link between theory and practice, transforming cognitive development into tangible competencies through actionable behaviors.

### 4.3 Limitations and future research

While this study establishes a theoretical framework linking supervisor support to graduate students’ critical thinking, several limitations should be acknowledged. The primary limitation is the sample’s focus on tourism management students within China’s supervisor responsibility system, which may affect the generalizability of the findings (Miao et al., 2025). To enhance external validity, future studies should recruit participants from a broader range of disciplines and investigate different supervisory contexts.

The second limitation pertains to the use of self-reported measures (Han et al., 2022; Zhou et al., 2025). Potential common method bias was assessed via confirmatory factor analysis (CFA), which indicated acceptable levels. Future studies should seek to validate these findings by incorporating data from multiple sources.

Third, the cross-sectional design of this study limits the ability to establish causal relationships between supervisor support and graduate students’ critical thinking (Han et al., 2022; Lee et al., 2026). As critical thinking is a dynamic developmental process, longitudinal research would better capture its progression and more rigorously examine the causal mechanisms linking supervisory factors to its trajectory.

## 5 Conclusion

First, supervisor support has a positive impact on graduate students’ critical thinking, a finding that supports the hypothesis. As the primary influence in students’ academic development, supervisors provide academic, personal, and autonomy support. By offering resources and assistance through research interactions, they grant students access to guidance and opportunities, which in turn enhances critical thinking.

Second, academic passion partially mediates the relationship between supervisor support and graduate students’ critical thinking—a finding that provides partial support for our hypothesis. This mediating mechanism is grounded in social information processing theory. The theory posits that individuals internalize supportive cues from their environment, transforming these cues into positive attitudes (i.e., academic passion), which in turn lead to enhanced cognitive outcomes. This passion is thus a key mediator. Specifically, meaningful supervisory support cultivates students’ intellectual curiosity, research enthusiasm, and intrinsic motivation for inquiry. These elements of passion, in turn, strengthen critical thinking.

Third, academic engagement partially mediates the relationship between supervisor support and graduate students’ critical thinking—a finding that provides partial support for the hypothesis. Consistent with social information processing theory, a supportive supervisory environment shapes student behavior, which in turn impacts outcomes. This supportive environment motivates learners to engage in analytical practice and knowledge application, thereby developing their critical thinking.

Fourth, academic passion and engagement play sequential mediating effect between supervisor support and critical thinking, a finding that supports the hypothesis Grounded in social information processing theory, it illustrates how environmental perceptions are transformed: first into attitudes, then into behaviors, and finally into enhanced outcomes. Specifically, this study delineates a sequential internalization process: supervisor support fosters critical thinking by first cultivating academic passion, which then motivates academic engagement, ultimately enhancing cognitive skills. Thus, the study delineates the internalization of external support—a transformative progression from attitude to behavior to advanced skill.

## Data Availability

The raw data supporting the conclusions of this article will be made available by the authors, without undue reservation.
